# A nationwide outbreak of listeriosis associated with cold-cuts, Sweden 2013-2014

**DOI:** 10.1080/20008686.2017.1324232

**Published:** 2017-06-13

**Authors:** Viktor Dahl, Lena Sundqvist, Ingela Hedenström, Margareta Löfdahl, Erik Alm, Håkan Ringberg, Mats Lindblad, Anders Wallensten, Susanne Thisted Lambertz, Cecilia Jernberg

**Affiliations:** ^a^ Department of Monitoring and Evaluation, The Public Health Agency of Sweden, Stockholm, Sweden; ^b^ European Programme for Intervention Epidemiology Training (EPIET), European Centre for Disease Prevention and Control, (ECDC), Stockholm, Sweden; ^c^ Department of Microbiology, The Public Health Agency of Sweden, Stockholm, Sweden; ^d^ Regional Office of Communicable Disease Control and Prevention, Region Skåne, Malmö, Sweden; ^e^ Food Control Department, The National Food Agency, Uppsala, Sweden; ^f^ Science Department, The National Food Agency, Uppsala, Sweden; ^g^ Department of Biomedical Sciences and Veterinary Public Health, Swedish University of Agricultural Sciences, Uppsala, Sweden

**Keywords:** Outbreak, *Listeria*, listeriosis, food, foodborne, Sweden

## Abstract

In January 2014, the Public Health Agency of Sweden noticed an increase in listeriosis cases. Isolates from 10 cases had identical pulsed field gel electrophoresis (PFGE) profiles, suggesting a common source. We investigated the outbreak to identify the source and stop transmission. We looked for cases in 2013–2014 and also compared cases notified after February 2014 to randomly selected controls. We surveyed food items consumed two weeks prior to symptom onset. *Listeria monocytogenes* isolates found by food producers were PFGE-typed. Patient and food isolates with the outbreak PFGE profile were whole-genome sequenced and 51 cases with identical PFGE profile were identified; 12/20 cases and 108/186 controls responded to the survey. All cases were exposed to cold-cuts, compared with 72% of controls (*p* = 0.034). Five isolates of *L. monocytogenes* with the outbreak PFGE profile were found in cold-cuts from a food producer which stopped production in February 2014, but cases appeared until October 2014. Whole-genome sequencing showed that cold-cut and patient isolates differed by eight single nucleotide polymorphisms. Three patient isolates differed more and were probably not part of the outbreak. Epidemiological and microbiological results indicated cold-cuts as a possible source of the outbreak.

## Introduction

*Listeria monocytogenes* is a foodborne bacterium that can infect both humans and animals.[1] Individuals with an impaired immune system (e.g. due to high age, pregnancy, high alcohol consumption, cancer or immune suppressive therapy) are at risk of severe invasive infection, with sepsis and/or meningitis.[2–4] In invasive infection, the median incubation period is 21 days (range 3–70 days). Invasive infection is treatable with antibiotics. In Sweden, the case fatality rate is 30% within three months after diagnosis, which includes mortality due to underlying conditions.[5] In 2012, the incidence of listeriosis in the European Union was 0.35 cases per 100,000 and in Sweden 0.60 cases per 100,000 (a total of 72 cases).[6,7] Between 1983 and 2012, the annual incidence of listeriosis increased in both Sweden and Europe, for unknown reasons.[5,8]

*Listeria monocytogenes* can grow at low temperatures. Low-level contamination during production can therefore reach infectious doses after storage of a food item at chilled temperatures, e.g. if the shelf-life is exceeded. The food items most commonly contaminated with *L. monocytogenes* during production are ready-to-eat foods made from meat, dairy, seafood and fresh produce.[9] A source attribution study in England and Wales identified multi-component foods such as sandwiches and pre-packed mixed salads as the most common sources of infection.[10] In 2010, a study in Sweden showed that *L. monocytogenes* was more prevalent in smoked and salt/sugar-cured (*gravad*) fish (12%) than in cold-cuts (1.2%) or soft cheese (0.4%).[11] The Swedish National Food Agency recommends that risk groups avoid consuming packaged, smoked or *gravad* fish and sliced cold-cuts that are not freshly produced. It also recommends that risk groups avoid mould-ripened and smear-ripened cheeses.[12] *Listeria monocytogenes* is a notifiable disease in Sweden, with clinicians and clinical microbiological laboratories reporting cases to the Public Health Agency of Sweden. Microbiological laboratories send all patient isolates to the Public Health Agency for typing.

In January 2014, the Public Health Agency of Sweden observed an increase in the number of notified cases of listeriosis at national level and also received calls from two regional offices that had made the same observation at county level. Ten cases with symptom onset during 2013 had identical PFGE profiles, suggesting a common source outbreak. On 14 January 2014 the Public Health Agency of Sweden therefore established an outbreak investigation team in collaboration with the National Food Agency in order to identify the source of the outbreak and put control measures in place.

## Materials and methods

### Listeria *isolates and epidemiological typing*

Isolates from notified cases of listeriosis in Sweden sent to the Public Health Agency for typing before 2015 were all serotyped (14) and further subtyped using pulsed field gel electrophoresis (PFGE) according to the PulseNet protocol,[13] with *Asc*I and *Apa*I as the restriction enzymes used. A transition to using whole genome sequencing (WGS) for typing started in 2014. In addition, from February 2014, food producers who found *L. monocytogenes* during routine controls were encouraged to send isolates to the National Food Agency for typing. There, the DNA band patterns from PFGE were analysed using the BioNumerics software (Applied Maths, NV; http://www.applied-maths.com/) and the Dice similarity coefficient and Ward’s method were used for cluster analysis. All *L. monocytogenes* isolates were also analysed using WGS. For this, barcoded libraries were prepared for the Ion Torrent 400 base-pair chemistry using Library Builder™ (Thermo Fischer Scientific, Waltham, MA, USA) and then the resulting libraries were pooled and size-selected using Pippin Prep™ (Sage Science, Los Angeles, CA, USA). The libraries were sequenced using Ion Torrent PGM™ (Thermo Fischer Scientific). The resulting sequence was quality trimmed and mapped to the reference genome. Variants were called using CLC Assembly Cell v 4.3.0 (Qiagen, https://www.qiagenbioinformatics.com/). The reference genome was constructed de novo from one of the outbreak isolates (2.92 Mbp, 287 contigs with N50 contig length of 28,432 bp and an average coverage of 36.7x), in order to capture variability in both the core and accessory genomes. Genomic positions for which more than one sample had coverage less than 20x were excluded. All other variable genomic positions (573 in the total dataset) were gathered into a multiple sequence alignment and 31 positions were eliminated from the analysis due to low coverage in one or more samples and 62 positions due to ambiguous calls in one or more samples, giving a final alignment size of 68 × 480. The average coverage for each sample varied between 23.7x and 99.9x. Minimum spanning trees were constructed using MSTgold v 2.4 (https://sourceforge.net/projects/mstgold/).

### Data collection and case definition

For each case, data on the variables age, sex, date of symptom onset, date of notification and place of residence were taken from the Swedish electronic (http://www.sminet.se/) system for notifiable diseases (SmiNet). Data on population size nationally and per county and on age and sex distribution were obtained from Statistics Sweden (www.scb.se). Exposures in the case-control study (described below) were determined by inviting cases and controls to answer a questionnaire either online or on paper.

Cases were defined as notified cases of listeriosis, suspected to have been contracted in Sweden, with the molecular serotype IIa and a specific PFGE outbreak profile using *Asc*I and *Apa*I between 1 January 2013 and 31 December 2014.

### Case control study

In order to identify exposures associated with being a case, cases that had been notified previously were compared to controls frequency-matched by age group (with a span of 10 years) and sex. These controls were recruited from a cohort representative of the Swedish population that was originally selected to receive weekly e-mail questionnaires on symptoms of respiratory and gastrointestinal infections for surveillance purposes. The controls were selected from this cohort using a list of randomly generated numbers. If insufficient controls were found in one age group, controls from the adjacent younger age group were sampled. The controls were invited to participate via an e-mail sent out 27 March 2014. The controls were interviewed in March and April 2014 about a period of exposure in March, but cases prior to February 2014 were not included because eating habits are different during the Christmas season. It was also suspected that cases that occurred before Christmas 2013 would not be able to recall food items consumed before developing symptoms, and they were therefore not included in the case-control study. Cases were considered to have been exposed to a food item if they had consumed it at least once within two weeks before symptom onset. Controls were considered to have been exposed to a food item if they had consumed it at least once within the two-week period prior to answering the questionnaire.

For the questions on consumption of a particular food item, the responses ‘Yes’ and ‘Probably’ were regarded as exposed and ‘Probably not’ and ‘No’ as non-exposed. The odds ratio of exposures was then calculated using STATA version 12 (Stata Statistical Software: Release 12, StataCorp. 2011). For proportions, a one-sample test was used for binomial proportion and normal-theory method (www.OpenEpi.com) to calculate a two-sided *p*-value.

## Results

### Descriptive epidemiology

**Figure 1.**
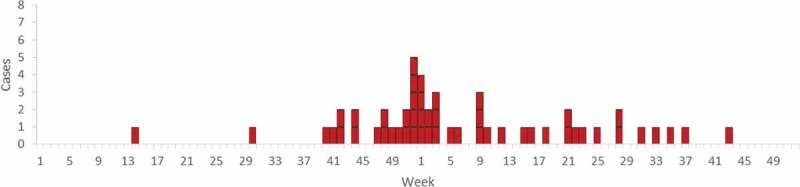
Distribution of cases of listeriosis with the outbreak PFGE profile over time. Each box represents one case (*N* = 51). Sweden, 2013–2014.

A total of 51 cases were identified during 2013–2014. The first case identified developed symptoms in week 14 (April) 2013, the outbreak peaked in week 52 (December) 2013 and the last case developed symptoms in week 43 (October) 2014 (Figure 1). Cases occurred in 16 of 21 Swedish counties. Of the 51 cases, 16 were from Skåne, the southernmost county (Table 1). The median age of cases was 77 years (range 28–98 years) and 82% of the cases were over 60 years (Table 2). Thirty-one cases (61%) were female, of which four were pregnant, and 20 cases (39%) were male (*p* = 0.12).

**Table 1.** Incidence per county of listeriosis with the outbreak PFGE profile, 2013–2014, Sweden.

County	Cases	Population	Incidence per 100,000
Gotlands län	2	57,161	3.5
Örebro län	5	285,395	1.8
Jämtlands län	2	126,461	1.6
Blekinge län	2	152,757	1.3
Skåne län	16	1,274,069	1.3
Kronobergs län	2	187,156	1.1
Kalmar län	2	233,874	0.9
Västra Götalands län	9	1,615,084	0.6
Västerbottens län	1	261,112	0.4
Värmlands län	1	273,815	0.4
Södermanlands län	1	277,569	0.4
Gävleborgs län	2	277,970	0.7
Hallands län	1	306,840	0.3
Uppsala län	1	345,481	0.3
Östergötlands län	1	437,848	0.2
Stockholms län	3	2,163,042	0.1
Dalarnas län	0	277,349	0.0
Jönköpings län	0	341,235	0.0
Norrbottens län	0	249,436	0.0
Västernorrlands län	0	242,156	0.0
Västmanlands län	0	259,054	0.0
Total	51	9,644,864	0.5

**Table 2.** Incidence per age group of listeriosis with the outbreak PFGE profile, Sweden, 2013–2014.

Age group (years)	Population	Cases	Incidence per 100,000
0–10	1,244,050	0	0.0
11–20	1,073,582	0	0.0
21–30	1,294,177	2	0.2
31–40	1,218,234	2	0.2
41–50	1,322,132	1	0.1
51–60	1,162,800	4	0.3
61–70	1,168,896	9	0.8
71–80	715,997	12	1.7
81–90	373,585	17	4.6
91+	71,411	4	5.6
Total	9,644,864	51	0.5

### Analytical epidemiology

Twenty cases were invited to participate in the case-control study and 12 (60%) responded to the questionnaire. Of 186 controls invited to participate, 108 (58%) responded to the questionnaire. Among controls, the response rate was higher among women (67%) than men (33%).

In univariate analysis, consumption of sliced smoked ham was associated with being a case (OR = 4.6, 95%CI 1.1–28). In all, 75% of cases had consumed this food item before developing symptoms (Supplementary Table 1).

Based on the results of the environmental investigation (described below), several exposures were combined in the analytical epidemiological study. It was found that 100% of cases were exposed to either sliced cooked ham, sliced smoked ham, sliced cooked *medwurst* sausage and/or liver pâté, compared with 72% of controls (*p* = 0.034). However, liver pâté was excluded from the combined variable since it is baked at a temperature that kills *L. monocytogenes* and it is usually not sliced after baking. Sliced cooked ham, sliced smoked ham and/or sliced cooked *medwurst* sausage was then associated with being a case (OR = 8.0, 95%CI 1.1–350), and 92% of cases were exposed.

### Laboratory and environmental investigation

On 8 February 2014, a major producer of cold-cuts announced that *L. monocytogenes* had been found during a routine control in sliced cooked *medwurst* sausage produced in its production plant on 8 January 2014. The company recalled all products sliced on that production line on 8 January 2014. The recalled products were cooked ham, smoked ham, cooked *medwurst* sausage and liver pâté. Isolates from samples of the initial products found to be positive for *L. monocytogenes* were discarded, so typing could not be performed. The company resumed production for commercial purposes after thorough cleaning of the production line and sampling of products and the environment before release, but then stopped again on 18 February when *L. monocytogenes* was found in sliced liver pâté, sliced smoked ham and sliced cooked *medwurst* sausage. At this time, the National Food Agency received isolates from environmental and food samples from the producer. Of 66 samples analysed, five were found to have a similar profile to the outbreak PFGE profile. The producer then closed the production line and dismantled it and slicing of cold-cuts was transferred to another production plant belonging to the same company.

Fifty-one patient isolates of the outbreak type were whole-genome sequenced and all isolates were found to have identical PFGE profiles. Furthermore, 10 historical isolates from 2010 and 2011 with the outbreak PFGE profile were sequenced. In addition to the five isolates from cold-cuts, seven isolates with the outbreak PFGE profile from other European countries in 2013 were also included. These originated from food (shrimp, brie, hamburgers and pork) and from swab samples from a slicing machine. One isolate was provided by the Federal Institute for Risk Assessment in Germany (BfR, Sylvia Kleta), and six by the Austrian Agency for Health and Food Safety (AGES, Ariane Pietzka). Whole-genome sequence data (raw reads) from one isolate from a Danish patient with the outbreak PFGE profile, diagnosed just prior to the Swedish outbreak, was added in the cluster analysis in order to determine whether that case was also part of the outbreak (provided by Jonas Larsson, Statens Serum Institut, Denmark).

**Figure 2.**
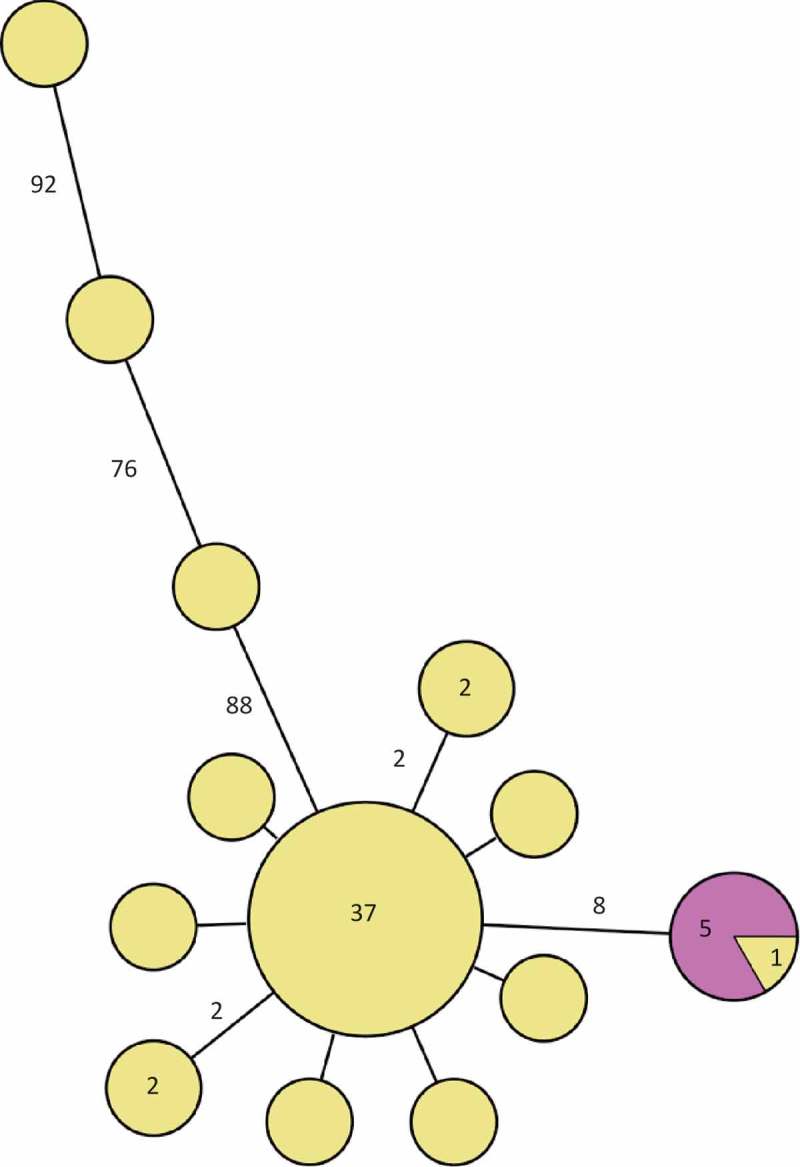
Minimum spanning tree of outbreak patient and food isolates (defined by PFGE as in the case definition). Each circle represents one isolate and each branch represents one single-nucleotide polymorphism (SNP) unless otherwise stated with a number within a circle or next to a branch. Patient outbreak isolates are shown in yellow and domestic food isolates from cold-cuts with the outbreak PFGE profile in purple.

**Figure 3.**
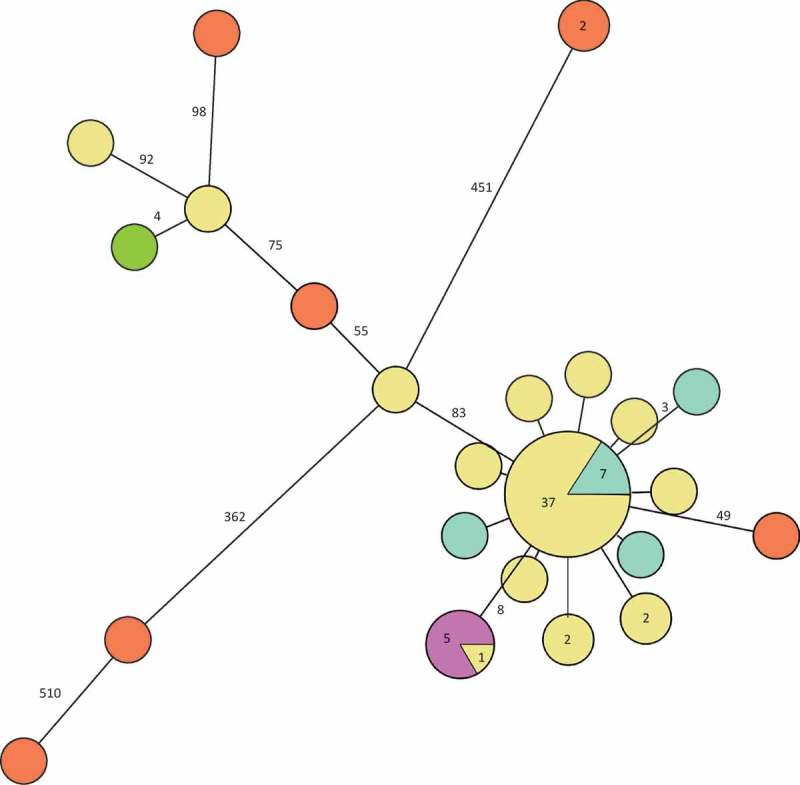
Minimum spanning tree of outbreak patient and food isolates (defined by PFGE as in the case definition) and also including historical Swedish patient isolates, a Danish patient isolate and European food isolates, all with the outbreak PFGE profile. Each circle represents one isolate and each branch represents one single-nucleotide polymorphism (SNP) unless otherwise stated with a number within a circle or next to a branch. Patient outbreak isolates are shown in yellow, Swedish patient isolates with the outbreak PFGE profile from previous years in turquoise, domestic food isolates from cold-cuts in purple, non-domestic food isolates in orange and the Danish patient isolate in green.

Of the Swedish 51 cases, isolates from 48 constituted the main cluster (separated by less than 10 single nucleotide polymorphisms (SNPs)) in the phylogenetic tree (Figure 2) and 37 of them were identical. Ten differed by one to two SNPs and three differed by more than 80 SNPs from the main outbreak cluster. The five isolates from the cold-cut producer were identical and clustered with one patient outbreak isolate. This cluster was separated by eight SNPs from the main cluster. Of the 10 Swedish historical patient isolates of the outbreak PFGE type, seven were identical to the main outbreak cluster and three differed by one to three SNPs (Figure 3). The seven non-domestic food and slicing machine isolates differed by more than 50 SNPs from the main cluster. One of the outliers of the outbreak patient isolates clustered with the Danish patient isolate.

## Discussion

Fifty-one cases in Sweden were identified as belonging to this outbreak (or 48 on excluding the three isolates that were not part of the outbreak according to WGS), making it one of the largest *L. monocytogenes* outbreaks ever described globally or in Europe. In North America, the largest reported outbreaks have been related to cantaloupe melons (USA 2011; 147 cases) [14] and ready-to-eat meat products (Canada 2008; 57 cases).[15] In Europe, large outbreaks have been linked to various types of cheese (Germany 2006–2007; 189 cases and Portugal 2009–2012: 30 cases) [16,17] and butter (Finland 1998–1999; 25 cases).[18] There is also an ongoing investigation of a large outbreak with an unknown source (Germany 2012–2015; 66 cases).[19]

The distribution of cases over time during the outbreak described in this paper suggests a persistent common source. There was a peak in the number of cases in the last week of 2013. The peak of the outbreak occurred approximately one incubation period after Christmas and could be due to higher consumption of the contaminated product during the Christmas season. The geographical distribution of cases was consistent with products from a company that distributes food nationally. The differences in incidence between Swedish counties could be due to chance, differences in the distribution of contaminated food items or differences in eating habits. The age distribution during the outbreak was similar to that usually seen for listeriosis in Sweden. Four of the cases were pregnant women and there are normally reports of 0–2 pregnant women with listeriosis per year in Sweden (unpublished data). This could indicate that the contaminated food item was not cheese or cured or smoked salmon, since these food items, to our experience, are avoided by many pregnant women in Sweden.

As *L. monocytogenes* with the outbreak PFGE profile was found in products from a production line where sliced cooked ham, sliced smoked ham, sliced cooked *medwurst* sausage and sliced liver pâté was produced, the hypothesis that all of these products were contaminated during production was tested. All cases had consumed at least one of these types of food items before developing symptoms, which was significantly more often than the controls. The producer, one of the major producers of cold-cuts in Sweden, recalled products and stopped production and moved slicing of cold-cuts to another production facility. However, cases continued to appear for eight months after the production line was closed. The shelf-life of cold-cuts is around three weeks, and the producer reported that it did not find *L. monocytogenes* in its internal control after the recall. Thus it seems unlikely that this production line was the only source of the outbreak. Cases reported after the production line had closed could hypothetically have been infected by cold-cuts stored in the refrigerator or freezer and consumed later, or could have had a prolonged incubation period, although this seems unlikely.

Other exposures that were significantly associated with being a case in our analytical study were frozen vegetables, hot-dogs and beetroot salad. However, all of these explained fewer cases than cold-cuts. Moreover, since hot-dogs and frozen vegetables are usually heated to a temperature that kills *L. monocytogenes* and since beetroot salad contains preservatives that prevent bacterial growth, they were all less likely to have been the source of infection.

On analysing the WGS data, it was found that three patient isolates with the outbreak PFGE profile were outliers in the phylogenetic tree. Two of these were also separated in time and occurred months before the others. This suggests that the outbreak started in October 2013 and that these two cases with the outbreak PFGE profile were unrelated to the outbreak.

The isolates with the outbreak PFGE profile that were sent to us by colleagues around Europe (food isolates, sequence of the Danish patient isolate) and included in the WGS for comparison, all differed genetically and were all outliers in the phylogenetic tree and therefore were not related to the Swedish outbreak. This shows that there can be considerable genetic diversity within the same PFGE profile and that the higher discriminatory power of WGS makes it an excellent tool in outbreak investigations.

Patient isolates from 2011 with a 0 SNPs difference from the main cluster could indicate that the outbreak was caused by a specific subtype that had been present in Sweden for some years. It could be widespread in Sweden and present at several different food producers, or it could have been present at the production plant for years and intermittently contaminated the food-producing surfaces. The persistence of a single subtype of *L. monocytogenes* for more than 10 years in a processing facility or on food processing surfaces has been reported previously.[20,21] However, there is also evidence to suggest that genetically very similar strains of *L. monocytogenes* can be found in multiple food-associated environments, indicating that genetic similarity does not necessarily mean a common source.[22]

Deciding the number of SNPs differences to use as a cut-off for case definition during an outbreak investigation is complex and more research on the genetic differences identified during an outbreak is needed. The cut-off will most probably also differ between different species and the environment in which they live. In addition, it will depend on the nature of the outbreak; some outbreaks are monoclonal, point source outbreaks and others, in particular listeriosis outbreaks, are prolonged, polyclonal, but from a single source. We found a difference of eight SNPs between the main patient cluster and the contaminated cold-cuts. This difference is slightly greater than that previously used for outbreak investigations for listeriosis using WGS. For example, in a listeriosis outbreak caused by ready-to-eat meat in Denmark in 2014, the cut-off was defined at ≤3 SNPs.[23] However, others have shown that *L. monocytogenes* strains known to be epidemiologically related can differ by up to 10 SNPs.[24] That study discusses the problem with setting absolute SNP thresholds for defining outbreak clusters and concludes that it is dependent on the reference genome selected (during our outbreak investigation we used one of the outbreak isolates), SNP calling parameters and sequencing metrics.[24]

This study had limitations. For example, all controls were interviewed about possible exposure in March 2014, while cases were interviewed throughout 2014, and eating habits might differ throughout the year. Moreover, two cases that were included in the case-control study according to the case definition based on PFGE were outliers in the phylogenetic analysis, and might not have been part of the outbreak.

## Conclusions

This case-control study indicated that the *L. monocytogenes* outbreak in Sweden 2013–2014 was associated with cold-cuts. *Listeria monocytogenes* with the outbreak PFGE profile was found in products from a production line where cold-cuts were produced, providing a microbiological link. The similarity of the patient isolates to the isolates found in the food items was confirmed by whole-genome sequencing for all but three cases. However, because the outbreak continued for another eight months after the production line was closed and slicing of cold-cuts was moved to another production plant, the association with this production facility remains uncertain.

## Public health actions

Based on the findings, we communicated current recommendations that cold-cuts that are not freshly produced should be avoided by risk groups, using the websites of the Public Health Agency of Sweden and the National Food Agency and through interviews in the media. Following this investigation, the National Food Agency performed official controls, including environmental sampling, at selected establishments producing cold-cuts during autumn and winter 2014. No samples with the outbreak PFGE profile were found during these controls.

## Recommendations

We recommend continuous typing of human isolates of *L. monocytogenes* in Sweden in order to identify clusters of cases. We encourage food producers to send all isolates of *L. monocytogenes* to the National Food Agency for typing, so that they can be compared with typing of human isolates, in order to rapidly identify the source of outbreaks. We recommend using whole-genome sequencing for molecular surveillance of listeriosis and in outbreak investigations. We also recommend that policy makers at the National Food Agency revise their recommendations and advise risk groups to avoid cold-cuts entirely.

## Supplementary Material

Supplementary Table 1
