# Supplementary material for: A nationwide outbreak of listeriosis associated with cold-cuts, Sweden 2013-2014
Source: Infect Ecol Epidemiol. 2017 Jun 13;7(1):1324232. doi: 10.1080/20008686.2017.1324232 (PMC5475333; doi:10.1080/20008686.2017.1324232)
Supplement: Supplementary Table 1 [file ziee_a_1324232_sm1413.docx]

|  |  | **Cases** |  |  | **Controls** |  |  |  |
| --- | --- | --- | --- | --- | --- | --- | --- | --- |
| **Exposure** | **Total** | **Exposed** | **%** | **Total** | **Exposed** | **%** | **OR [95% CI]** | **p** |
| Boiled ham, smoked ham, *medwurst* (a sliced boiled sausage) or liverwurst | 12 | 12 | 100 | 114 | 82 | 72 | **. [1.1-.]** | 0.034 |
| Boiled ham, smoked ham or *medwurst* | 12 | 11 | 92 | 112 | 65 | 58 | **8.0 [1.1-350]** |  |
|  |  |  |  |  |  |  |  |  |
| Smoked ham | 12 | 9 | 75 | 109 | 43 | 39 | **4.6 [1.1-28]** |  |
| Liverwurst | 12 | 7 | 58 | 111 | 44 | 40 | 2.1 [0.54-9.0] |  |
| Boiled ham | 12 | 6 | 50 | 110 | 31 | 28 | 2.6 [0.62-10] |  |
| Salami | 12 | 4 | 33 | 108 | 28 | 26 | 1.4 [0.29-5.8] |  |
| Other type of meat paste | 11 | 4 | 36 | 105 | 14 | 13 | 3.7 [0.69-17] |  |
| *Medwurst* | 12 | 3 | 25 | 110 | 18 | 16 | 1.7 [0.27-7.72] |  |
| Hamburger meat (horse meat) | 12 | 2 | 17 | 107 | 6 | 6 | 3.4 [0.29-22] |  |
| Any type of meat spread | 12 | 12 | 100 | 111 | 88 | 79 | . [0.79-.] |  |
|  |  |  |  |  |  |  |  |  |
| Shrimp salad | 12 | 5 | 42 | 110 | 16 | 15 | 4.2 [0.9-17] |  |
| Potatoe salad | 12 | 3 | 25 | 109 | 10 | 9 | 3.3 [0.49-16] |  |
| Beetroot salad | 11 | 3 | 27 | 109 | 4 | 4 | **9.8 [1.2-68]** |  |
| Other type of creamy salad | 10 | 2 | 20 | 104 | 2 | 2 | 13 [0.78-190] |  |
| Tuna salad | 12 | 1 | 8 | 111 | 1 | 1 | 10 [0.12-790] |  |
| Chicken salad | 12 | 1 | 8 | 108 | 2 | 2 | 4.8 [0.07-97] |  |
| Mimosa salad | 11 | 1 | 9 | 111 | 4 | 4 | 2.7 [0.05-31] |  |
|  |  |  |  |  |  |  |  |  |
| Mixed frozen vegetables | 11 | 7 | 64 | 103 | 13 | 13 | **12 [2.6-62]** |  |
| Hot dogs | 11 | 7 | 64 | 104 | 14 | 13 | **11 [2.4-58]** |  |
